# Ethical considerations in rapid and novel treatments in psychiatry

**DOI:** 10.1038/s41386-023-01635-y

**Published:** 2023-06-30

**Authors:** Tobias Haeusermann, Winston Chiong

**Affiliations:** UCSF Weill Institute for Neurosciences, University of California, San Francisco, CA USA

**Keywords:** Depression, Drug development

## Abstract

New treatment modalities for mental illness are deeply needed, and emerging therapeutic agents such as psychedelics, ketamine, and neuromodulatory technologies have been welcomed by many researchers and patients. These treatment approaches have also been observed to raise novel ethical questions, and to pose new and different versions of familiar ethical questions in clinical treatment and research. We present an overview and introduction to these issues organized around three specific domains of ethical concern: informed consent, the role of expectancy in clinical response, and distributive justice.

There is growing interest among patients, clinicians, researchers, and the broader public in the prospect of novel, rapid-acting therapeutics for psychiatric disorders—such as, though not limited to, psychedelics, ketamine and other NMDA receptor antagonists, and new neuromodulatory technologies. This interest is informed by acknowledged limitations of conventional antidepressant drug treatments (such as therapeutic lag and a high proportion of clinical non-response), concern over the growing public health impact of mental illness, greater societal openness in discussing mental health, and the potential of new research to yield theoretical contributions to our understanding of the human brain [1]. While some have heralded these novel interventions as the beginning of a new era in psychiatry (or even of a broader social transformation), enthusiasm must be balanced by caution given the complexities that these novel interventions raise at multiple levels—not only the psychological and physiological, but also political, cultural, and historical.

One set of issues raised by these novel therapeutics concerns the relationship between clinical and research ethics, as researchers may be perceived as gatekeepers of interventions that are strongly desired by people with mood disorders and other psychiatric disorders. Whereas some treatments are already available through off-label prescriptions (i.e ketamine [2]), most novel and rapid treatments are legally or practicably restricted to the realm of clinical studies (e.g., LSD, psilocybin, ayahuasca, closed-loop neuromodulation) [3, 4]. We present an overview and introduction to special ethical considerations arising together in clinical care and research with novel psychiatric interventions, with references to more detailed discussions for the interested reader. The ethical concerns raised by such interventions are particularly wide-ranging. We have organized this overview around discussions of informed consent, the role of expectancy in clinical response, and distributive justice; as these topics may help to highlight unique features of these novel and rapid-acting treatments that raise special concern.

## Informed consent

A fundamental challenge in psychiatric and neurologic care and research is that the conditions of interest may directly interfere with cognitive processes necessary for consent to be valid and informed. Of course, clinicians and researchers should be aware that capacity is determined on an individual case-by-case basis, and not solely on categorical grounds such as having a particular diagnosis or deficit. In contemporary clinical practice, standards for consent to treatment identify four essential abilities that underlie capacity to consent: understanding, appreciation, reasoning, and the expression of choice [5, 6]. “Understanding” indicates a patient’s or research participant’s ability to grasp the essential elements of their medical decision—including the nature of their condition, the recommended treatment or proposed research study, and its potential risks and benefits. “Appreciation” indicates a person’s ability to apply these elements to their own case. “Reasoning” refers to the ability to logically manipulate information, such as weighing their treatment’s likely outcomes against those of other treatment modalities. Finally, capacity requires the ability to “express a choice,” which, besides articulating a treatment or research participation preference requires a relative stability of that same preference (in the absence of new information prompting changes) [7, 8]. It must be added, however, that individuals who fall short of the full range of decisional capacities should not be excluded from research and care. We therefore suggest that clinicians and researchers duly consider participants’ retained abilities and preferences when making such determinations, as well as other accommodations and protections such as seeking support from a surrogate decision-maker or the enrollment of caregivers as study partners. Moreover, though assessing patient capacity for consent is a central tenet of bioethics, it often directs attention to individual participants’ abilities when the social and institutional contexts of their decisions are often just as critical. Some scholars have offered a multidimensional “functional account” of informed consent that accommodates layered aspects of the role that consent conversations play in research decisions and research protections [9]. Beyond concerns about capacity, rapid and novel psychiatric treatments may require additional disclosures beyond what is ordinarily encompassed in consent conversations. Some treatments raise questions about increased out-of-pocket expenses and access to continuing care; for instance, in the case of implanted neuromodulatory devices, provisions for device explantation or for continuing device support if a participant benefits from the intervention (even if the study outcome is negative at a group or statistical level) [10]. A further specific concern about rapid and novel treatments in psychiatry is that in some cases these agents may induce wider-ranging changes in political or metaphysical commitments extending beyond intended therapeutic effects [11]. As these possibilities are quite removed from standard biomedical risks often considered in consent documents, how to incorporate them in “enhanced” consent conversations has remained challenging [12].

## The role of expectancy in clinical response

Establishing consent is an important first step towards the delivery of ethical care and research, but further steps are needed. At this stage of development, rapid and novel treatments are intended for individuals with major psychiatric symptoms who do not respond to first-line treatments. Given that diagnostic and trial inclusion criteria for many trials require a failure of established therapies, the threshold to participation might be lowered and significantly alter individuals’ risk-benefit evaluation. With desperation and hope comes a heightened level of vulnerability, amplified by reports of “miracle cures” and “breakthroughs”. When exploring influences on potential subjects’ decision making in deep brain stimulation (DBS) research for depression, Christopher et al. identified hoped-for improvements, perceived lack of other treatment options and a desire to take initiative as influential driving forces among participants [13]. Whereas every treatment is inherently affected by placebo and nocebo effects, psychiatric therapies present a particularly complex case [14]. With psychotropic drugs, for instance, outcome expectancies have shown remarkably strong effects relative to the drugs’ understood physiologic mechanisms [15]; methodological challenges are often amplified when patient-reported outcomes are the primary efficacy measures [16]. In trials of novel and rapid treatment, greater in-treatment patient expectancy is tied to greater depressive symptom reduction - yet may thereby also increase patient-participants’ vulnerability. It is therefore particularly important for clinicians and researchers to convey the differing objectives, goals and expectations of research and treatment – with both their short and long-term implications - and assume their moral responsibility to minimize the risk of harming their patients [17]. Keeping in mind some agents’ potentially addictive properties and the attendant risk for misuse, rapid and novel treatments should therefore be incorporated within overall, long-lasting therapeutic plans and outcome measures, often also incorporating non-pharmacologic interventions such as psychotherapy or other psycho-social interventions.

An additional complexity specifically relates to fast-acting and *experience-altering* therapies. Some rapid and novel treatments induce drastic alterations in consciousness, presenting deep methodological challenges for the application of traditional blinded, randomized controlled trial designs [12, 18].The mechanism of action of the therapeutic effects of many novel treatments are unknown, but these alterations in consciousness may be causally important to their mechanism—in which case the use of an active placebo that alters consciousness to a comparable degree may mask true observed effects. Combining active placebos with alternative trial designs (e.g., dose-response parallel-groups design) along with intentional vagueness during the consent process regarding the treatment’s acute effects have been offered as recommendations [19], but these approaches present their own ethical as well as logistical concerns.

## Community and structure

As noted above, bioethical analysis tends to direct attention towards individual-level considerations such as consent capacity, but the questions posed by novel and rapid psychiatric treatments also present challenges at the level of community and the broader structure in which psychiatric care and research are delivered—particularly with regard to distributive justice. For example, while researchers and clinicians should be cognizant of potential patient-participant vulnerabilities as described above, the aim of protecting socioeconomically vulnerable (or otherwise structurally vulnerable) individuals can often result in exclusion from societally important and often personally desired research participation. Historically, participants in clinical depression trials have not been demographically representative of the broader society. Effects across racial and ethnic groups are underreported and linguistic minority groups are often largely excluded [20].

Insufficient consideration of diversity not only hampers studies’ generalizability but also leaves unaddressed the specific needs and concerns of more vulnerable or marginalized groups [21, 22], potentially perpetuating historic inequities and existing neurodisparities [23]. Ongoing research indicates coming challenges in delivering new brain-based treatments, including problems of equity and access, in ensuring that benefits of research are available to those in greatest need of new approaches [24]. In many settings, patients may struggle with limited access, community resources, and time for therapy/research [25]. Clinical trials and, ultimately, implementation for rapid and novel treatments ought to be guided by inclusion of diverse patient and caregiver voices, which will also require screening tools to be translated as well as culturally adapted and locally validated.

Lastly, some novel treatments have a long and rich history of medicinal, recreational, and ceremonial use; as such, their incorporation into biomedicine raises concerns of justice and about cultural appropriation. In the ongoing translational evolution of these treatments, this history merits respect and acknowledgment, as important lessons can be drawn from previous experiences. This is likely to entail involving representatives of indigenous communities and others that have traditionally been excluded from the research process, as well as new conceptualizations of our models for scientific discovery and validation.
