# Correction: Ethical considerations in rapid and novel treatments in psychiatry

**DOI:** 10.1038/s41386-023-01669-2

**Published:** 2023-07-28

**Authors:** Tobias Haeusermann, Winston Chiong

**Affiliations:** UCSF Weill Institute for Neurosciences, University of California, San Francisco, CA USA

**Keywords:** Depression, Drug development

Correction to: *Neuropsychopharmacology* 10.1038/s41386-023-01635-y, published online 30 June 2023

The original online version of this article was revised: In the sentence “Whereas some treatments are already available through off-label prescriptions (i.e R-ketamine [2]), most novel and rapid treatments are legally or practicably restricted to the realm of clinical studies (e.g., LSD, psilocybin, ayahuasca, closed-loop neuromodulation) [3, 4]”, ‘R-ketamine’ was corrected to ‘ketamine’. The original article has been corrected.

